# A Multicenter, Prospective, Observational Study to Assess the Clinical Activity and Impact on Symptom Burden and Patients’ Quality of Life in Patients with Advanced Soft Tissue Sarcomas Treated with Trabectedin in a Real-World Setting in Greece

**DOI:** 10.3390/cancers14081879

**Published:** 2022-04-08

**Authors:** Stefania Kokkali, Ioannis Boukovinas, Epaminondas Samantas, Pavlos Papakotoulas, Ilias Athanasiadis, Charalampos Andreadis, Parisis Makrantonakis, Georgios Samelis, Eleni Timotheadou, Georgios Vassilopoulos, Christos Papadimitriou, Dimitrios Tzanninis, Alexandros Ardavanis, Ioannis Kotsantis, Kiki Karvounis-Marolachakis, Theodora Theodoropoulou, Amanda Psyrri

**Affiliations:** 1 First Department of Medical Oncology, Agios Savvas Athens General Hospital, 11522 Athens, Greece; ardavanis@yahoo.com; 2 Medical Oncology, Bioclinic of Thessaloniki, 54622 Thessaloniki, Greece; ibouk@otenet.gr; 3 Third Oncology Clinic, Agioi Anargiroi Athens General Hospital, 14564 Athens, Greece; epsam@otenet.gr; 4 First Chemotherapeutic Oncology Department, Theagenion Anti-Cancer Hospital of Thessaloniki, 54639 Thessaloniki, Greece; papakotoulas@gmail.com; 5 Oncology Department, Hygeia Athens Private Hospital, 15123 Maroussi, Greece; iathanasiadis@hygeia.gr; 6 Third Department of Clinical Oncology and Chemotherapy, Theagenion Anti-Cancer Hospital of Thessaloniki, 54639 Thessaloniki, Greece; andreadisc@gmail.com; 7 Second Department of Clinical Oncology and Chemotherapy, Theagenion Anti-Cancer Hospital of Thessaloniki, 54639 Thessaloniki, Greece; pmakrant@gmail.com; 8 Oncology Department, Hippocration General Hospital of Athens, 11527 Athens, Greece; gsamelis@otenet.gr; 9 Department of Medical Oncology, Papageorgiou Hospital, Faculty of Medicine, School of Health Sciences, Aristotle University of Thessaloniki, 54629 Thessaloniki, Greece; timotheadou@auth.gr; 10 Department of Hematology, Larissa University Hospital, 41334 Larissa, Greece; gvasilop@bioacademy.gr; 11 Oncology Unit, Aretaieion University Hospital, National and Kapodistrian University of Athens, 11528 Athens, Greece; chr_papadim@yahoo.gr; 12 Medical Oncology Unit, Athens Medical Center, 15125 Maroussi, Greece; j.janinis@gmail.com; 13 Division Medical Oncology, Attikon University General Hospital of Athens, 12462 Haidari, Greece; ikotsantis@gmail.com (I.K.); psyrri237@yahoo.com (A.P.); 14 Medical Department, Genesis Pharma SA, 15232 Halandri, Greece; kkarvouni@genesispharma.com (K.K.-M.); ttheodoropoulou@genesispharma.com (T.T.)

**Keywords:** advanced soft tissue sarcoma, observational, quality of life, real-world, trabectedin

## Abstract

**Simple Summary:**

Soft tissue sarcomas (STS) constitute a group of heterogeneous tumors. For patients with advanced or metastatic disease, prognosis is poor and only a few treatments are available, including trabectedin. The aim of our prospective multicenter study was to evaluate the real-world activity of trabectedin, and its impact on symptom burden and quality of life in patients with advanced STS treated in routine clinical settings in Greece. Between 21 December 2015 and 6 June 2018, 64 eligible patients from 13 Greek centers were evaluated. Our study provides real-world evidence on the effectiveness, tolerability and safety of trabectedin in a population of patients with advanced STS of multiple histological subgroups who have either experienced a relapse or disease progression after standard-of-care front-line therapy, or were unsuited to receive front-line agents.

**Abstract:**

This non-interventional, multicenter, prospective study aimed to evaluate the real-world activity of trabectedin, and its impact on symptom burden and quality of life in patients with advanced soft tissue sarcoma (aSTS) treated in routine clinical settings in Greece. Patients with histologically confirmed aSTS newly initiated on trabectedin were enrolled. The primary endpoint was progression-free survival (PFS) rate at 6 months. Secondary endpoints included PFS rate at 3 months, median PFS, objective response rate (ORR), disease control rate (DCR), overall survival (OS), and an assessment of the impact of treatment on health-related quality of life (HRQoL), cancer-related symptom burden and symptom interference with function, as well as all-cause treatment discontinuation rate. A total of 64 eligible patients from 13 Greek centers were evaluated. Patients received a median of three trabectedin cycles per patient (interquartile range [IQR]: 2.0–6.0). Median PFS was 6.6 months with 67.9% and 51.2% of patients free from progression at 3 and 6 months, respectively. ORR was 7.8% and DCR 21.9%. Median OS was 13.1 months. No significant changes from enrolment were noted in HRQoL scores. In total, 30 patients (46.9%) had at least one trabectedin-related adverse drug reaction (ADR) and 9 (14.1%) at least one serious ADR. The treatment discontinuation rate due to toxicity was 9.4%. These results suggest that trabectedin is an active treatment with clinically meaningful benefits in patients with aSTS with no new safety signals.

## 1. Introduction

Soft tissue sarcomas (STS) constitute a group of heterogeneous tumors of mesenchymal origin, classified into more than 80 histological types [1]. STS are rare, accounting for less than 1% of adult malignancies [2]. The incidence of STS in Europe averages 3–5/100,000 annually [3,4,5,6] and the estimated 5-year survival rate among patients of any type and stage at diagnosis is higher than 50% [3,7,8]. However, for patients with advanced or metastatic disease, prognosis is poor, with a median overall survival (OS) of approximately 1.5 years from the start of therapy [9], along with health-related quality of life (HRQoL) deterioration with disease progression [10,11].

Treatment decision-making in STS is guided by several disease parameters, such as histology and clinical stage, as well as the performance status (PS) of the patient [1,12]. For locally advanced or metastatic STS, sequential use of doxorubicin-based chemotherapy and ifosfamide as single agents or a combination of these agents is the mainstay approach in Europe [1,9]. Response rates with these treatment options range from 21% to 56% [9], but their prolonged use is limited by drug-related toxicity [13].

Trabectedin (Yondelis^®^; PharmaMar, S.A., Madrid, Spain) is a semisynthetic drug originally isolated from the sea squirt Ecteinascidia turbinate [14]. Trabectedin is the first anticancer marine-derived drug approved in the European Union in 2007 for patients with advanced STS (aSTS) after failure of anthracyclines or ifosfamide, as well as for those who are unsuited to receive such agents, which serves as a treatment option in the second-line setting and beyond [1,15]. The efficacy and safety of trabectedin has been assessed in several phase II and III trials, demonstrating a clinical benefit in terms of progression-free survival (PFS) and disease control rate (DCR) in patients with aSTS [16,17,18] and particularly in those with advanced liposarcoma or leiomyosarcoma (commonly abbreviated as L-sarcomas) [19,20,21]. Trabectedin treatment is also feasible in non-L-sarcomas as it has demonstrated efficacy in patients with a variety of histologically different sarcoma subtypes [22,23].

Following the approval of trabectedin, the majority of real-world evidence in Europe derives from single-center studies [23,24,25,26,27,28] and mainly conducted as retrospective analyses [23,24,25,26,27,28,29,30,31,32,33]. In contrast, prospective, multicenter studies of trabectedin in patients with aSTS in routine clinical practice are generally lacking [34,35]. Therefore, the aim of the “BEYOND-STS” multicenter, prospective, non-interventional study was to assess the clinical effectiveness and safety of trabectedin as well as its impact on cancer-related symptoms and patients’ HRQoL in an unselected and a more diverse real-world population of patients with aSTS across Greece.

## 2. Materials and Methods

### 2.1. Study Design

This was a non-interventional, multicenter, prospective cohort study which included patients with aSTS initiated on trabectedin under routine clinical practice in Greece and given in accordance with the marketing authorization. The study was carried out by hospital-based oncologists specializing in sarcoma care and practicing in geographically diverse locations across Greece. Consistent with the real-world observational nature of the study, there was no involvement with any treatment decisions for the patients included in the study as they were treated without any additional per protocol instructions. The choice of therapy had to be made prior to the patient’s inclusion in the study.

The study consecutively enrolled, over an approximate 2.5-year accrual period, adult patients (≥18 years old) with a histologically confirmed diagnosis of aSTS (locally advanced or metastatic), who had failed treatment with anthracyclines and ifosfamide or who were unsuited to receive these drugs. Patients who had received more than one cycle of trabectedin, as well as patients who were receiving or had recently received treatment with any investigational product (within 1 month or 5 half-lives of the investigational drug, whichever was longer) were excluded from participation. 

Primary data were collected by physicians both generated according to routine clinical practice and reported by the patients through patient-reported outcomes (PROs) at 6-week intervals (corresponding to every 2 cycles of treatment with trabectedin) during the first 24 weeks of therapy and every 12 weeks thereafter for up to 182 weeks. Treated patients were followed-up until the last patient enrollment plus up to 54 weeks of treatment or until disease progression, death, withdrawal of consent, unacceptable toxicity, study completion, or physician’s decision (whichever occurred earlier). Patients who discontinued treatment were followed for up to 24 weeks post-treatment discontinuation. 

This study was designed and conducted in accordance with the principles of the International Society for Pharmacoepidemiology guidelines for Good Pharmacoepidemiology Practice, the ethical principles laid down in the Declaration of Helsinki, the STROBE (Strengthening the Reporting of Observational Studies in Epidemiology) guidelines where applicable, and all applicable local rules and regulations. Patients were included in the study after signing an informed consent form (ICF).

### 2.2. Study Objectives

The study’s primary objective was to evaluate the effectiveness of treatment with trabectedin by assessing progression-free survival rate (PFSR) at 6 months post-treatment initiation. Secondary effectiveness objectives included the evaluation of the 3-month PFSR, median PFS, objective response (ORR) and disease control (DCR) rates, and overall survival (OS). In addition, the study assessed the impact of trabectedin on HRQoL, cancer-related symptom burden and symptom interference with function, as well as the all-cause treatment discontinuation rate. 

### 2.3. Endpoint Definitions and Assessments

Tumor response to trabectedin was evaluated by the participating physicians according to local and institutional common practice and Response Evaluation Criteria in Solid Tumors (RECIST) v1.1 [36]. PFS was defined as the time from the date of treatment initiation until the first radiographic documentation of objective tumor progression or death regardless of cause. OS was defined as the interval between treatment initiation and the date of death regardless of cause or the date of last follow-up. ORR was defined as the proportion of patients with either a radiological complete response (CR) or a partial response (PR) as best objective response. DCR was defined as the proportion of patients achieving an ORR or stable disease (SD) for at least 24 weeks (i.e., ≥24 weeks have elapsed from first to last response assessment or the patient had been on treatment for at least 24 weeks). 

The impact of treatment with trabectedin on the cancer-related symptom burden and symptom interference with function was assessed through the use of the Greek-validated version of M.D. Anderson Symptom Inventory (G-MDASI), whereas HRQoL was assessed using the Greek-validated version of EuroQoL 5-Dimensions, 3-Levels (EQ-5D-3L) questionnaire. The PROs were collected via self-administered questionnaires completed by the patients.

Safety data were collected from enrolment until 30 days post-treatment discontinuation. All AEs were coded using the Medical Dictionary for Regulatory Activities (MedDRA), v.22.1 and graded according to the National Cancer Institute-Common Terminology Criteria (NCI-CTC), v.5.0.

### 2.4. Treatments

Trabectedin was administered in accordance with the local marketing authorization and the treating clinician’s discretion depending on the patient’s conditions and previous chemotherapy. The recommended dose of trabectedin for the treatment of STS is 1.5 mg/m^2^ body surface area, administered as an intravenous infusion over 24 h with a 3-week interval between cycles. Pretreatment with corticosteroids (e.g., dexamethasone 20 mg intravenously 30 min before trabectedin) was considered mandatory for all patients receiving trabectedin. 

There were no predefined limits to the number of trabectedin cycles administered and treatment could continue as long as the treating physician judged there was clinical benefit, even in the presence of apparent disease progression in target disease, or consent withdrawal. Once trabectedin treatment was discontinued, patients could have been treated with subsequent anticancer therapies or supportive care as per the clinician’s clinical judgment.

### 2.5. Sample Size Determination

The sample size calculation was based on the study’s primary endpoint. According to available published data at the time of designing this study, the 6-month PFSR for patients with aSTS treated with trabectedin ranged between 24 and 39% [16,17,18,19,37]. Taking into account the real-world design of the study and the fact that patients were expected to have less favorable clinical characteristics, an assumption of a 30% PSFR was made. Based on this assumption, a sample size of 90 patients was considered adequate to estimate the aforementioned rate with a precision of ±9.5% (95% confidence interval [CI]: 21.5–39.5%). Taking into account a non-evaluable rate of 10%, a final sample size of 100 patients was proposed, in order to ensure the aforementioned sample size for the final statistical analysis.

### 2.6. Statistical Analysis Methods

Descriptive analyses to summarize patient, disease, and treatment characteristics were performed with appropriate statistical methods (i.e., median, minimum, and maximum for continuous variables or interquartile range [IQR]; numbers and percentages for categorical variables). The 3- and 6-month PFSRs, PFS, and OS, along with the respective 95% CIs were estimated according to the Kaplan–Meier method. For PFS, patients who were alive and progression-free at the time of study completion were censored 30 days post-treatment discontinuation, while for OS analysis, patients who were alive were censored at their last follow-up date. The association of patient and disease characteristics of interest with PFS was examined by univariable Cox regression analysis. The ORR and DCR were calculated along with the respective 95% CIs. With regard to the evaluation of the change in G-MDASI, EQ-5D (UK) Index and EQ-VAS scores from enrolment to study-predefined subsequent timepoints, the Wilcoxon signed rank sum test was used. Mean differences (SD) and *p*-values were calculated for paired data. All *p*-values were descriptive in nature and all statistical tests were two-sided and have been performed at a 0.05 significance level. Statistical analyses were conducted using the SAS^®^ (version 9.4) statistical analysis software.

## 3. Results

### 3.1. Patient Disposition and Characteristics

From 21 December 2015 (first patient first visit; FPFV) to 06 June 2018 (last patient last visit; LPLV), 66 patients signed the ICF. Of these, two (3.0%) patients were excluded from study participation as they did not fulfill all eligibility criteria. Thus, 64 eligible patients, who were enrolled by 13 public and private oncology hospital centers/clinics, comprised the full analysis set.

All patients were Caucasian and 62.5% were female (Table 1). At enrolment, the patients’ median age was 58.3 years, most (93.8%) had ECOG PS ≤ 1, and 53.1% had ≥1 comorbidity. The most frequent comorbidity reported was ‘cardiovascular disease other than coronary artery disease (CAD)’ (16 patients, 25.0%), followed by ‘thyroid disorders’ (9 patients, 14.1%), and ‘CAD’ (5 patients, 7.8%).

The median time from initial STS diagnosis to study enrolment was 15.3 (IQR: 7.8–35.6) months. A total of 13patients (20.3%) had been diagnosed with aSTS at initial presentation. 

Among enrolled patients, L-sarcomas were the most prevalent histological subtypes (32, 50%), (Table 1). Lower extremity was the most common site of STS (27, 42.2%). The majority of patients had metastatic disease (43, 67.2%) with the lung being the most prominent site of distant metastasis in around 75% of patients. Among patients with available data, most primary tumors were of size > 5 cm (34/53, 64.2%), while 43.8% (21/46) were poorly differentiated. 

Prior to trabectedin treatment initiation, 82.8% of the patients had received chemotherapy; 53.1% had received only one, and 29.7% at least two prior treatment lines, respectively (Table 1). In the first-line setting, the agents used at a frequency greater than 10% among patients with available data (*n* = 52) were anthracyclines (doxorubicin and/or epirubicin) (*n* = 33, 63.5%), ifosfamide (*n* = 29, 55.8%), docetaxel (*n* = 8, 15.4%), and gemcitabine (*n* = 7, 13.5%), with the combination of doxorubicin with ifosfamide being the most commonly administered regimen (*n* = 14, 26.9%). In the second-line setting, the most frequently used agents were pazopanib (*n* = 9, 47.4%); docetaxel, doxorubicin, and gemcitabine (*n* = 3, 15.8% each); and ifosfamide, cyclophosphamide, and vincristine (*n* = 2, 10.5% each).

### 3.2. Treatment

All patients were initiated on trabectedin at the recommended dose of 1.5 mg/m^2^ body surface area (BSA) administered as an intravenous infusion over 24 h every 3weeks. A total of 18 (28.1%) patients had initiated trabectedin prior to enrolment. The last treatment dose was 1.5, 1.2 and 1.0 mg/m^2^ BSA for 49 (76.6%), 12 (18.8%) and 3 (4.7%) patients, respectively. 

Further, 15 patients (23.4%) experienced 25 AEs that led to dose reduction. Of these, 13 patients had hematologic toxicities (anemia grade 2 (*n* = 1), anemia grade 3 (1), anemia grade 4 (3), neutropenia grade 3 (5), pancytopenia grade 4 (2), and thrombocytopenia grade 4 (1)). Other events leading to dose reductions included fatigue grade 3 (*n* = 4), nausea grade 2 (1), hepatitis grade 3 (1), hepatotoxicity grade 3 (1), hypertransaminasaemia grade 3 (1), pyrexia grade 2 (1), rhabdomyolysis grade 4 (1), toxicity to various agents (1), and unclear reason (1). All events, except for pyrexia, were related to trabectedin treatment. Trabectedin administration was withheld at least once during the study in 10 (15.6%) patients. 

In the end, 63 patients permanently discontinued treatment with trabectedin, due to disease progression (PD) (*n* = 27, 42.2%), patient’s choice (10, 15.6%), physician’s decision (8, 12.5%), and death/toxicity/lost to follow-up for 6 (9.4%) patients each. 

Over a median treatment duration of 1.8 (IQR: 0.8–3.9) months (mean: 3.7 (standard deviation, SD: 4.6) months), the median number of trabectedin cycles received was 3 (IQR: 2.0–6.0; range 1–27); 45 patients (70.3%) received ≥3 cycles, 19 (29.7%) ≥ 6 cycles, and 11 (17.2%) ≥ 12 cycles. At the end of study, one patient (1.6%) was still on treatment and had received 24 cycles.

### 3.3. Effectiveness

**Figure 1:**
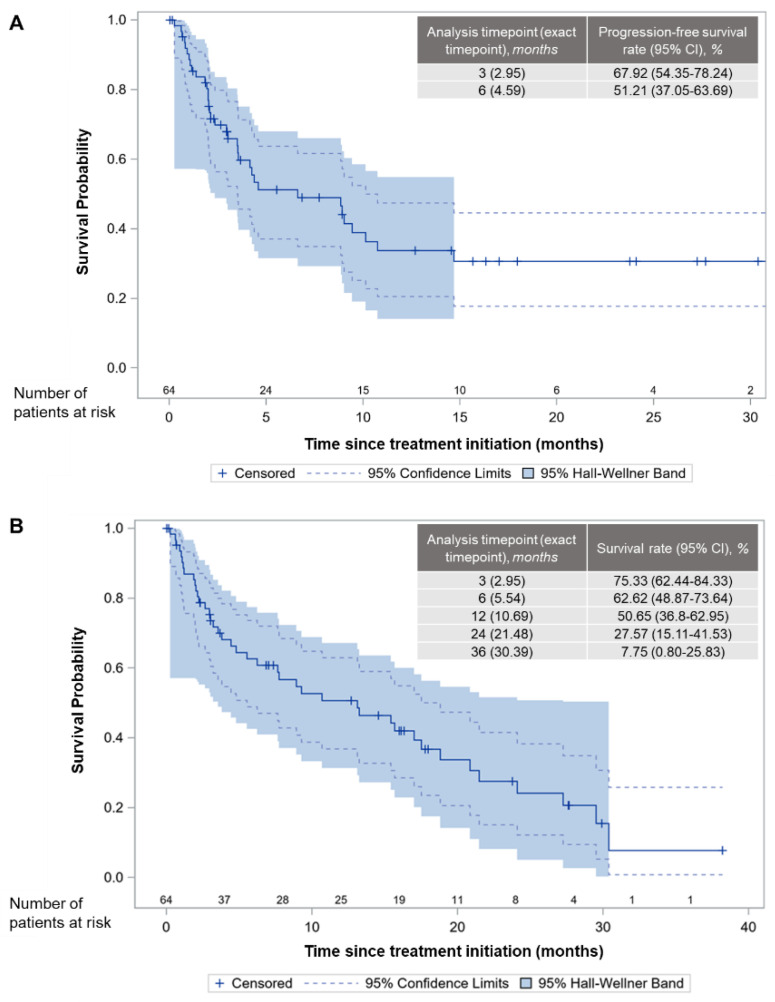
Kaplan–Meier curves and landmark time analysis in the full analysis set. (**A**) progression-free survival. (**B**) overall survival.

At the data cut-off date for PFS analysis, 29 patients were alive and progression-free (Table 2). Median PFS was 6.6 (95% CI: 3.5–10.1) months, whereas the 3- and 6-month PFSRs after treatment initiation were 67.9% (95% CI: 54.4%–78.2%) and 51.2% (95% CI: 37.1%–63.7%), respectively (Figure 1A).

Based on the univariable analysis of baseline factors that may have an impact on PFS, no statistically significant association with PFS was found with the following variables: ‘age’, ‘gender’, ‘pattern of STS extent’, ‘lung metastasis’, ‘prior surgery’ and ‘prior radiotherapy’. In contrast, ECOG PS score of ≥1 at enrolment was significantly associated with an increased risk of disease progression/all-cause death as compared with PS score of 0 (HR: 2.69; *p* = 0.002) (Table 3).

At the end of the study (i.e., LPLV), 23 (35.9%) of the patients were alive (Table 4). With a median time on study of 6.9 (IQR: 2.3–16.7; range, 0.0–38.2) months, median OS was 13.1 (95% CI: 5.5–18.8) months, whereas the OS rates at various time points are shown in Figure 1B. The ORR and DCR were 7.8% (95% CI: 1.2–14.4%) and 21.9% (95% CI: 11.8–32.0%), respectively, with five patients (7.8%) achieving PR, and nine (14.1%) patients achieving stable disease.

Among the 41 (64.1%) deceased patients, reasons for death were PD (36), treatment-related toxicity (1), acute peritonitis (1), cardio-respiratory deficiency (1), malignant disease (1), and pulmonary embolism (1). 

In total, by the end of the study or the last follow-up visit, PD was documented for 38 (59.4%) patients; the most common site of progression was the lung (*n* = 21) followed by the liver (*n* = 8), while all other sites were reported for ≤4 patients each.

### 3.4. HRQoL

At enrolment, 52 (81.3%) patients completed the G-MDASI and EQ-5D questionnaires. For the G-MDASI subscale regarding symptom severity, ‘fatigue’ and ‘pain’ were the items with the highest (worst) mean scores of 3.1 (SD: 3.0) and 2.2 (SD: 2.9), respectively (Supplementary Figure S1A). For the G-MDASI subscale regarding interference with daily life, ‘walking’ and ‘working’ were the items with the highest (worst) mean scores of 3.0 (SD: 3.4) and 2.9 (SD: 3.5), respectively. Based on the patient-reported EQ-5D-3L outcomes, the most affected dimensions at enrolment were ‘pain/discomfort’ and ‘anxiety/depression’, in which 29 (55.8%) and 27 (51.9%) patients reported problems, respectively (Supplementary Figure S1B).

**Figure 2:**
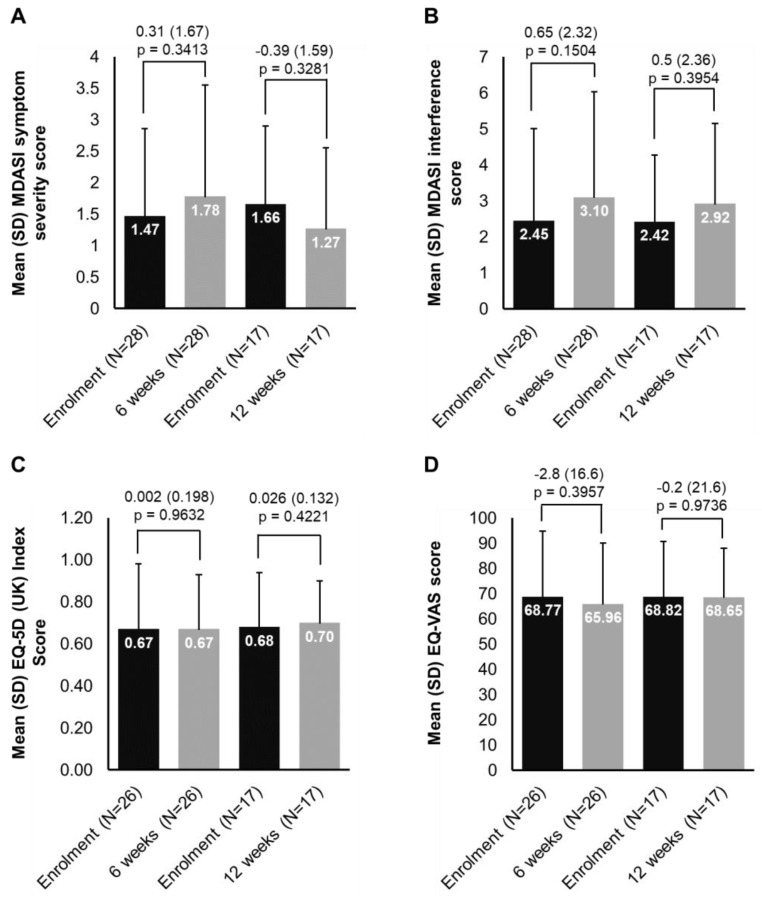
Changes in PROs from enrolment at week 6 and 12: (**A**) MDASI symptom severity, (**B**) MDASI interference, (**C**) EQ-5D (UK) Index, (**D**) EQ-VAS. Each of the 13 symptoms in the MDASI symptom severity scale is rated on an 11-point scale to indicate the presence and severity of the symptom in the last 24 h with 0 meaning ‘not present’ and 10 meaning ‘as bad as you can imagine’. Each of the 6 items in the MDASI symptom interference scale is rated based on the level of symptom interference with the function of a patient’s life in the last 24 h and also measured on an 11-point scale (0 = ‘did not interfere’ to 10 = ‘interfered completely’). The EQ-5D-3L descriptive system comprises 5 dimensions and each dimension is rated based on 3 levels: no problems, some problems, extreme problems. The EQ-VAS records the respondent’s self-rated health on a vertical, visual analog scale (gradated from 0–100) where the endpoints are labeled ‘Best imaginable health state’ and ‘Worst imaginable health state’ with higher scores indicating higher HRQoL.EQ-5D—EuroQoL 5-Dimensions; MDASI—M.D. Anderson Symptom Inventory; N—number of patients; PRO—patient-reported outcome; SD—standard deviation; UK—United Kingdom; VAS—Visual Analogue Scale.

The scores per PRO item for each timepoint are illustrated in Supplementary Figure S1. Figure 2 illustrates mean scores at enrolment, at 6 and 12 weeks in patients with available paired data for G-MDASI, EQ-5D (UK) Index Score and EQ-VAS, as well as the differences in the scores over the treatment period. The number of patients who completed the questionnaires is also depicted, which significantly decreased at 12 weeks.

### 3.5. Safety

During the safety data collection period, 51 (79.7%) patients experienced at least one AE, 25 (39.1%) at least one serious AE, 30 (46.9%) at least one trabectedin-related AE (adverse drug reaction, ADR), and 9(14.1%) at least one serious ADR (Table 5). Of these, 12(18.8%) patients experienced AEs leading to trabectedin discontinuation. Two events assessed as being related to trabectedin had fatal outcome.

## 4. Discussion

The “BEYOND-STS” study provides real-world evidence on the effectiveness, tolerability, and safety of trabectedin in a population of patients with aSTS of multiple histologies who have either experienced a relapse or disease progression after standard-of-care front-line therapy, or were unsuited to receive front-line agents. Patients were enrolled with non-limiting eligibility criteria apart from those indicated by the European authorization of trabectedin [15]. Effectiveness was assessed based on PFS and OS outcomes, while the impact of trabectedin on HRQoL was evaluated using PROs.

The majority of patients in the “BEYOND-STS” study received trabectedin in the second-or-laterline of therapy, in line with most other real-world data on trabectedin use in Europe [23,24,25,26,27,28,29,30,31,32,33,34]. Treatment with trabectedin resulted in a median PFS of 6.6 months, which is higher than the 1.6 to 4.2 month range reported across clinical trials [16,18,19,20], but comparable to the 2.2 to 7.5 month range reported by other real-world studies in Europe [23,25,26,27,28,29,31,32,33,34,37]. The PFSR at 6 months in the present study reached 51.2%, confirming drug activity [38], while the respective rate at 3 months was 67.9%. These rates are also higher than clinical trial findings, reporting PFSR in the range of 24%–37% [16,17,18,19,20] at 6 months and 39%–56% [16,17,19,20] at 3 months, and in line with most real-world trabectedin studies in Europe, where 6-month PFS rates range between 37%–49% [27,29,33,34,37] and 3-month rates between 58%–70% [27,33,34].

Differences in PFS among studies could be due to several factors, including patient and disease characteristics. For instance, consistent with other studies [24,30], Cox regression analysis presented herein supports that a better performance status at baseline was associated with better PFS. In contrast, though in line with the limited published literature, this study showed that baseline demographic and disease or clinical characteristics of age [23,24,26,30,37], gender [23,24,26,30,37], STS extent of disease [24], and lung metastasis [37] did not affect the outcome of trabectedin on PFS. Moreover, no significant association was identified in “BEYOND-STS” between PFS and prior surgery or radiotherapy.

“BEYOND-STS” also assessed tumor response to trabectedin per RECIST and as per common clinical practice. The observed 7.8% ORR and 21.9% DCR were low compared to previously published real-world data on tumor response to trabectedin in Europe (4%–33% [23,24,25,26,27,29,30,31,32,33,34,37] and 44%–77.5% [23,24,25,26,27,29,30,31,33,34,37] for ORR and DCR, respectively). It is likely that variations in the duration of the observation period, the response assessment criteria used, as well as patient and disease characteristics may have contributed to this difference. Disease histology has been shown to impact efficacy outcomes [21,27,29,32,33], with undifferentiated pleomorphic sarcoma (UPS) being associated with low response rates [29]. Compared with other real-world trabectedin studies (3.7%–10.4%) [26,29,30,34], in “BEYOND-STS” UPS was overrepresented (15.6%). Importantly, in most real-world studies, a requirement with regards to the length of stable disease in order to qualify for disease control was either not defined [23,24,26,27,29,30,31,33,34,37], or was much less than 6 months (i.e., 3 months) [25]. On the other hand, the DCR observed in “BEYOND-STS” is comparable to the rates (24%–34%) reported in clinical trials where a similar DCR definition was employed (i.e., duration of stable disease for 18–24 weeks) [16,17,20].

In “BEYOND-STS”, a median OS of 13.1 months was reported, which compares well with historical clinical trial data (9.2–15.8 months) [16,17,18,19,21]. In the real-world setting, although longer median OS has been reported by some studies, the range across Europe is quite wide (7–23.5 months) [23,25,26,27,28,29,31,32,33,34,37]. Differences in reported median OS could be attributable to several factors such as variations in length of follow-up and distribution of histological subtypes [27,29,30,31,33]. In our study, after discontinuation of treatment (*n* = 63), patients were followed-up for 24 weeks and thus mortality status was not controlled after that time. Indicatively, in studies reporting a median OS of >20 months [26,29,34] cohorts seem to have a higher distribution of L-sarcomas (62.5–68%) than the present cohort (50.0%). Furthermore, differences in median OS could also reflect the variation in the number of trabectedin cycles received across studies. For instance, in the prospective, non-interventional multicenter phase IV study “Y-IMAGE”, reporting a median OS of 21.3 months, the median cycle number (6) and percentage of patients receiving ≥6 cycles (56.9%) were almost double than those observed in “BEYOND-STS” (3 and 30%, respectively). More generally, studies where patients received a median of 3–4 cycles report median OS of 7–16.5 months [23,27,28,31,32,33,37], while studies where patients received 4–9 cycles of trabectedin report median OS in the range of 19.3–23.5 months [25,26,29,34] and data support that aSTS patients treated for ≥6 cycles have a better prognosis [37].

Besides effectiveness outcomes, the present study addressed the effect of trabectedin treatment on aSTS patients’ HRQoL. We intended to assess if there was a difference in symptom burden and QoL over the study period. However, we were not able to draw any conclusion, given the small number of patients who responded at subsequent timepoints (especially at week 12). Although 52 patients responded at baseline, only 28 and 17 responded at 6 and 12 weeks respectively, mainly due to the fact that many patients had discontinued treatment. In this limited analysis, MDASI and EQ-5D scores did not decline substantially with trabectedin treatment, in line with MDASI clinical trial data on trabectedin [39] and symptoms of ‘pain’, ‘asthenia’, and ‘feeling’ in the prospective, non-interventional study “Y-IMAGE” [34]. At present, HRQoL data for STS are scarce, mostly stemming from cross-sectional studies investigating QoL [11] and symptom burden at therapy onset [40,41]. However, the incorporation of HRQoL measurements in clinical trials and daily practice is becoming an increasingly important focus [35], as QoL deterioration is known to occur as the disease progresses [10,11], and its delay remains one of the main goals of treatment for aSTS.

With respect to safety, treatment-related AEs observed in “BEYOND-STS” primarily affected the hematopoietic system, consistent with the unwanted consequences of chemotherapy. Overall, the safety profile of trabectedin in “BEYOND-STS” was in agreement with that reported in phase II and III clinical trials [16,17,18,19,20,21] as well in a series of real-world studies in Europe [23,25,26,27,29,32,33,34], with no unexpected safety signals arising. Consistent with clinical trial and real-world safety data [16,17,18,20], the most frequent reason for trabectedin treatment discontinuation in “BEYOND-STS” was disease progression. Furthermore, 9.4% of the patients discontinued treatment due to toxicity, which lies within the range reported in other clinical studies, i.e., 0–18% [16,17,18,19,20,25,27,28,29,31,34], while the 3.1% rate of deaths related to trabectedin observed herein is also in agreement with previous studies [16,17,19,20,21,25,31,33].

Limitations in our study are mainly attributable to its observational design, lack of central pathology review by an expert pathologist, missing data, lack of internal control, patient information bias and a limited sample size. Only patients who agreed to participate were registered, provoking a potential selection bias. To assure that the study population accurately reflects the characteristics of the Greek aSTS population, it would be useful to analyze also the non-participants during the same period. Even though physicians were encouraged to employ the same assessment technique and criteria over the study period, response assessment was not centrally reviewed, hence may be influenced by observer bias. Loss to follow-up bias must also be kept in mind when interpreting the results presented herein, as this could not be accounted for due to the lack of a control group. In addition, although the chosen PRO instruments did not involve a recall period (EQ-5D-3L) or employed a short-term recall period (24 h for G-MDASI), recall bias may have been introduced for certain patients who had received the first dose prior to enrolment and were asked to complete the questionnaires based on their health status at the time of treatment initiation. The overall impact of treatment on patient reported outcomes cannot be assessed, due to the limited number of subjects with available data over time.

Finally, the limited sample, which did not reach the originally planned size, did not allow for an extensive analysis on associations of treatment outcome with patient and disease characteristics, and may have impacted the precision of the estimation of the primary outcome measure. However, considering the rarity of the disease [3,4,5,6], and that the proportion of patients who will go on to receive second- or third-line therapy for advanced or metastatic disease is even lower [9], this sample size has a good semblance to the aSTS population treated with trabectedin in Greece. Patient recruitment from 13 different oncology centers, of the private and public sector, located in different geographic regions also enhances the generalizability of the findings for the country. Therefore, the results of “BEYOND-STS” provide an overview of the patient’s characteristics, trabectedin use, and outcomes in routine clinical practice in Greece.

## 5. Conclusions

In conclusion, the findings of this real-world, multicenter, prospective study showsthat trabectedin provides a clinically meaningful benefit in terms of clinical outcomes, in aSTS patients who have failed or are unsuited to receive anthracycline/ifosfamide.

## Figures and Tables

**Table 1:** Patient and disease characteristics at enrolment.

Characteristic	Full Analysis Set (*n* = 64)
Age, median (IQR)	58.3 (46.9–64.8)
Gender, *n* (%)	
Female	40 (62.5)
Male	24 (37.5)
Educational level, *n* (%)	
No education	7 (10.9)
Primary education	14 (21.9)
Secondary education	26 (40.6)
Tertiary education	17 (26.6)
Employment status, *n* (%)	
Unemployed	10 (15.6)
Employed	27 (42.2)
Retired	13 (20.3)
Household duties	11 (17.2)
Other	3 (4.7)
BMI classification, *n* (%)	
<18.5	4 (6.3)
18.5–24.9	24 (37.5)
25.0–29.9	23 (35.9)
≥30.0	13 (20.3)
PS (ECOG), *n* (%)	
0	38 (59.4)
1	22 (34.4)
2	2 (3.1)
3	2 (3.1)
Presence of at least one comorbidity, *n* (%)	34 (53.1)
Histological Subtype	*n* (%)
Leiomyosarcoma	21 (32.8)
UPS	10 (15.6)
Liposarcoma	7 (10.9)
Myxoid Liposarcoma	4 (6.3)
Synovial Sarcoma	3 (4.7)
Unclassified	3 (4.7)
Fibrosarcoma	2 (3.1)
Other ^1^	14 (21.9)
Metastasis sites, *n* (%)	43 (67.2)
Lung	32 (50.0)
Bone	7 (10.9)
Liver	7 (10.9)
Nodes	5 (7.8)
Pelvis	4 (6.3)
Brain	1 (1.6)
Other	7 (10.9)
Prior surgery, *n* (%)	26 (40.6) ^2^
Complete tumor excision, *n*/*n* (%)	12 (18.9)
Prior radiotherapy, *n* (%)	15 (23.4) ^2^
Number of prior lines of chemotherapy, *n* (%)	
0	11 (17.2)
1	34 (53.1)
2	16 (25.0)
3	3 (4.7)

^1^ High-grade sarcoma lipomatous and fibrous (*n* = 1), malignant peripheral nerve sheath tumor (4), myxoinflammatory fibroblastic sarcoma (1), clear cell sarcoma (2), epithelioid sarcoma (2), embryonal rhabdomyosarcoma (1), fibromyxoid sarcoma (1), malignant solitary fibrous tumor (1), and myeloid sarcoma (1). ^2^ Prior surgery and prior radiotherapy were unknown for one and three patients, respectively.aSTS—advanced STS; BMI—body mass index; ECOG—Eastern Cooperative Oncology Group; IQR—interquartile range; N—number of patients; PS—Performance Status; SD—standard deviation; STS—soft tissue sarcoma; UPS—Undifferentiated Pleomorphic Sarcoma.

**Table 2:** Progression-free survival analysis.

Number	Death or PD Events	Censored (30 Days Post-Treatment Discontinuation)
*n*	35	29

PD: progressive disease.

**Table 3:** Univariable Cox regression analysis of PFS.

Variable	Hazard Ratio (95% CI)	*p*-Value
Age (Years)	1.01 (0.98–1.03)	0.5888
Gender (Male vs. Female)	0.81 (0.42–1.57)	0.5357
STS extent (Locally advanced vs. Metastatic)	0.76 (0.36–1.560)	0.4659
ECOG PS (1–3 vs. 0)	2.69 (1.43–5.05)	0.0021
Lung metastasis (Yes vs. No)	0.97 (0.52–1.81)	0.9317
Prior surgery (Yes vs. No)	1.47 (0.76–2.83)	0.2508
Prior radiotherapy (Yes vs. No)	0.68 (0.34–1.38)	0.2888

CI—confidence interval; ECOG—Eastern Cooperative Oncology Group; PFS—progression-free survival; PS—Performance Status; STS—soft tissue sarcoma.

**Table 4:** Overall survival analysis.

Number	Death Events	Censored (at the Last Follow-Up Visit)
*n*	41	23

**Table 5:** Trabectedin-related adverse events.

Trabectedin-Related AEs	Full Analysis Set (*n* = 64)				
	Overall		Serious	Grade 1–2	Grade 3–4
	n_events_	n_pt_ (%)	n_pt_ (%)	n_pt_ (%)	n_pt_ (%)
Trabectedin-related AEs in ≥2% of patients (>1 patient)	87	30 (46.9)	9 (14.1)	20 (31.3)	18 (28.1)
Fatigue	17	14 (21.9)	1 (1.6)	4 (6.3)	10 (15.6)
Anemia	17	11 (17.2)	5 (7.8)	9 (14.1)	4 (6.3)
Neutropenia	8	6 (9.4)	-	3 (4.7)	4 (6.3)
Nausea	6	6 (9.4)	-	6 (9.4)	-
Thrombocytopenia	5	5 (7.8)	2 (3.1)	3 (4.7)	2 (3.1)
Vomiting	5	5 (7.8)	-	4 (6.3)	1 (1.6)
Leukopenia	3	3 (4.7)	-	3 (4.7)	-
Febrile neutropenia	2 ^1^	2 (3.1)	2 (3.1)	-	1 (1.6)
Hepatitis	2 ^1^	2 (3.1)	2 (3.1)	-	1 (1.6)
Pancytopenia	2	2 (3.1)	2 (3.1)	-	2 (3.1)

Treatment-related AEs occurring in <2% of patients included: grade 1–2—non-serious events of abdominal pain, constipation, diarrhea, dizziness, dysgeusia, flatulence, hypersomnia, lymphopenia, peripheral sensory neuropathy, and thrombotic thrombocytopenic purpura occurring in one patient each (1.6%); grade 3–4—non-serious events of hypertransaminasaemia and toxicity to various agents occurring in one patient each (1.6%); grade 3–4—serious events of hepatotoxicity, pneumonia, pyrexia, and rhabdomyolysis occurring in 1 patient each (1.6%). ^1^ One event of febrile neutropenia and one event of hepatitis, experienced by 1 patient each, had fatal outcomes. AE—adverse event; nevents—number of events; npt—number of patients with ≥1 event of the respective category.

## Data Availability

ClinicalTrials.gov Identifier: NCT02618122 (https://clinicaltrials.gov/ct2/show/NCT02618122, accessed on 20 December 2021). The data presented in this study are available on request from the corresponding author, due to restrictions e.g., privacy or ethical, following approval from the Study Sponsor. Due to privacy and ethical restrictions, raw data remain confidential and are not to be shared.

## Supplementary Materials

The following are available online at https://www.mdpi.com/article/10.3390/cancers14081879/s1, Figure S1: PRO item scores at baseline, week 6 and week 12.

## References

[1] Casali P.G., Abecassis N., Aro H.T., Bauer S., Biagini R., Bielack S., Bonvalot S., Boukovinas I., Bovee J.V.M.G., Brodowicz T. (2018). Soft tissue and visceral sarcomas: ESMO-EURACAN Clinical Practice Guidelines for diagnosis, treatment and follow-up. Ann. Oncol. Off. J. Eur. Soc. Med. Oncol..

[2] National Cancer Institute SEER Cancer Stat Facts: Soft Tissue Cancer. https://seer.cancer.gov/statfacts/html/soft.html.

[3] Stiller C.A., Trama A., Serraino D., Rossid S., Navarro C., Chirlaquee M.D., Casali P.G., The RARECARE Working Group (2013). Descriptive epidemiology of sarcomas in Europe: Report from the RARECARE project. Eur. J. Cancer.

[4] Gatta G., Van Der Zwan J.M., Casali P.G., Siesling S., Dei Tos A.P., Kunkler I., Otter R., Licitra L., Mallone S., Tavilla A. (2011). Rare cancers are not so rare: The rare cancer burden in Europe. Eur. J. Cancer.

[5] Rare Cancer Network in Europe. http://rarecarenet.istitutotumori.mi.it/.

[6] Robinson D., Nersesyan K., Pomerantz D. (2015). Epidemiology And Treatment Of Soft Tissue Sarcoma In The Eu5. Value Health.

[7] Cancer Research UK Soft Tissue Sarcoma. https://www.cancerresearchuk.org/about-cancer/soft-tissue-sarcoma/survival.

[8] Stiller C.A., Botta L., Brewster D.H., Ho V.K.Y., Frezza A.M., Whelan J., Casali P.G., Trama A., Gatta G., EUROCARE-5 Working Group (2018). Survival of adults with cancers of bone or soft tissue in Europe-Report from the EUROCARE-5 study. Cancer Epidemiol..

[9] Nagar S.P., Mytelka D.S., Candrilli S.D., D’yachkova Y., Lorenzo M., Kasper B., Lopez-Martin J.A., Kaye J.A. (2018). Treatment Patterns and Survival among Adult Patients with Advanced Soft Tissue Sarcoma: A Retrospective Medical Record Review in the United Kingdom, Spain, Germany, and France. Sarcoma.

[10] Eichler M., Hentschel L., Richter S., Hohenberger P., Kasper B., Andreou D., Pink D., Jakob J., Singer S., Grützmann R. (2020). The Health-Related Quality of Life of Sarcoma Patients and Survivors in Germany-Cross-Sectional Results of a Nationwide Observational Study (PROSa). Cancers.

[11] Reichardt P., Leahy M., Garcia del Muro X., Ferrari S., Martin J., Gelderblom H., Wang J., Krishna A., Eriksson J., Staddon A. (2012). Quality of Life and Utility in Patients with Metastatic Soft Tissue and Bone Sarcoma: The Sarcoma Treatment and Burden of Illness in North America and Europe (SABINE) Study. Sarcoma.

[12] NCCN Clinical Practice Guidelines in Oncology, Soft Tissue Sarcoma, Version 1.2021, 30 October 2020. https://www.nccn.org/guidelines/guidelines-detail?category=1&id=1464.

[13] Wesolowski R., Budd G.T. (2010). Use of chemotherapy for patients with bone and soft-tissue sarcomas. Clevel. Clin. J. Med..

[14] Cuevas C., Francesch A. (2009). Development of Yondelis (trabectedin, ET-743). A semisynthetic process solves the supply problem. Nat. Prod. Rep..

[15] European Medicines Agency Yondelis® Summary of Product Characteristics. Last Updated on 9 October 2020. https://www.ema.europa.eu/en/documents/product-information/yondelis-epar-product-information_en.pdf.

[16] Yovine A., Riofrio M., Blay J.Y., Brain E., Alexandre J., Kahatt C., Taamma A., Jimeno J., Martin C., Salhi Y. (2004). Phase II Study of Ecteinascidin-743 in Advanced Pretreated Soft Tissue Sarcoma Patients. J. Clin. Oncol..

[17] Le Cesne A., Blay J.Y., Judson I., Van Oosterom A., Verweij J., Radford J., Lorigan P., Rodenhuis S., Ray-Coquard I., Bonvalot S. (2005). Phase II study of ET-743 in advanced soft tissue sarcomas: A European Organisation for the Research and Treatment of Cancer (EORTC) soft tissue and bone sarcoma group trial. J. Clin. Oncol. Off. J. Am. Soc. Clin. Oncol..

[18] Garcia-Carbonero R., Supko J.G., Maki R.G., Manola J., Ryan D.P., Harmon D., Puchalski T.A., Goss G., Seiden M.V., Waxman A. (2005). Ecteinascidin-743 (ET-743) for chemotherapy-naive patients with advanced soft tissue sarcomas: Multicenter phase II and pharmacokinetic study. J. Clin. Oncol. Off. J. Am. Soc. Clin. Oncol..

[19] Demetri G.D., Chawla S.P., von Mehren M., Ritch P., Baker L.H., Blay J.Y., Hande K.R., Keohan M.L., Samuels B.L., Schuetze S. (2009). Efficacy and safety of trabectedin in patients with advanced or metastatic liposarcoma or leiomyosarcoma after failure of prior anthracyclines and ifosfamide: Results of a randomized phase II study of two different schedules. J. Clin. Oncol. Off. J. Am. Soc. Clin. Oncol..

[20] Demetri G.D., Mehren M., Jones R.L., Hensley M.L., Schuetze S.M., Staddon A., Milhem M., Elias A., Ganjoo K., Tawbi H. (2016). Efficacy and Safety of Trabectedin or Dacarbazine for Metastatic Liposarcoma or Leiomyosarcoma After Failure of Conventional Chemotherapy: Results of a Phase III Randomized Multicenter Clinical Trial. J. Clin. Oncol. Off. J. Am. Soc. Clin. Oncol..

[21] Patel S., Mehren M., Reed D.R., Kaiser P., Charlson J., Ryan C.W., Rushing D., Livingston M., Singh A., Seth R. (2019). Overall survival and histology-specific subgroup analyses from a phase 3, randomized controlled study of trabectedin or dacarbazine in patients with advanced liposarcoma or leiomyosarcoma. Cancer.

[22] Reichardt P., Grünwald V., Kasper B., Schuler M., Gelderblom H. (2015). Efficacy of trabectedin in patients with some rare advanced soft tissue sarcoma subtypes other than liposarcoma and leiomyosarcoma. J. Med. Drug Rev..

[23] De Sanctis R., Marrari A., Marchetti S., Mussi C., Balzarini L., Lutman F.R., Daolio P., Bastoni S., Bertuzzi A.F., Quagliuolo V. (2015). Efficacy of trabectedin in advanced soft tissue sarcoma: Beyond lipo- and leiomyosarcoma. Drug Des. Dev. Ther..

[24] Fabbroni C., Fucà G., Ligorio F., Fumagalli E., Barisella M., Collini P., Morosi C., Gronchi A., Tos A.P., Casali P.G. (2021). Impact of Pathological Stratification on the Clinical Outcomes of Advanced Well-Differentiated/Dedifferentiated Liposarcoma Treated with Trabectedin. Cancers.

[25] Gounaris I., Hatcher H.M., Davidson D., Sherbourne K., Alam S., Zaki K.A., Horan G., Earl H.M. (2014). Trabectedin for advanced soft tissue sarcomas: A single institution experience. Future Oncol..

[26] Hindi N., García I.C., Sánchez-Camacho A., Gutierrez A., Peinado J., Rincón I., Benedetti J., Sancho P., Santos P., Sánchez-Bustos P. (2020). Trabectedin Plus Radiotherapy for Advanced Soft-Tissue Sarcoma: Experience in Forty Patients Treated at a Sarcoma Reference Center. Cancers.

[27] Schmitt T., Keller E., Dietrich S., Wuchter P., Ho A.D., Egerer G. (2010). Trabectedin for metastatic soft tissue sarcoma: A retrospective single center analysis. Mar. Drugs.

[28] Schur S., Lamm W., Köstler W.J., Hoetzenecker K., Nemecek E., Schwameis K., Klepetko W., Windhager R., Brodowicz T. (2013). Trabectedin in patients with metastatic soft tissue sarcoma: A retrospective single center analysis. Anti-Cancer Drugs.

[29] Palmerini E., Sanfilippo R., Grignani G., Buonadonna A., Romanini A., Badalamenti G., Ferraresi V., Vincenzi B., Comandone A., Pizzolorusso A. (2021). Trabectedin for Patients with Advanced Soft Tissue Sarcoma: A Non-Interventional, Retrospective, Multicenter Study of the Italian Sarcoma Group. Cancers.

[30] Martínez-Trufero J., Sande-González L.M., Luna P., Martin-Broto J., Álvarez R., Marquina G., Beveridge R.D., Poveda A., Cano J.M., Cruz-Jurado J. (2021). A Growth Modulation Index-Based GEISTRA Score as a New Prognostic Tool for Trabectedin Efficacy in Patients with Advanced Soft Tissue Sarcomas: A Spanish Group for Sarcoma Research (GEIS) Retrospective Study. Cancers.

[31] Schack L.H., Mouritsen L.S., Elowsson C., Krarup-Hansen A., Safwat A. (2015). The Danish experience with trabectedin treatment for metastatic sarcoma: Importance of hyponatremia. Acta Oncol. (Stockh. Swed.).

[32] Ploner F., Lamm W., Schur S., Eisterer W., Kühr T., Lindorfer A., Tinchon C., Köstler W.J., Szkandera J., Brodowicz T. (2013). The Austrian experience with trabectedin in non-selected patients with metastatic soft tissue sarcoma (STS). J. Cancer Res. Clin. Oncol..

[33] Le Cesne A., Ray-Coquard I., Duffaud F., Chevreau C., Penel N., Nguyen B.B., Piperno-Neumann S., Delcambre C., Rios M., Chaigneau L. (2015). Trabectedin in patients with advanced soft tissue sarcoma: A retrospective national analysis of the French Sarcoma Group. Eur. J. Cancer.

[34] Buonadonna A., Casanova C.B.J., Kasper B., Pousa A.L., Mazzeo F., Brodowicz T., Penel N. (2017). A noninterventional, multicenter, prospective phase IV study of trabectedin in patients with advanced soft tissue sarcoma. Anti-Cancer Drugs.

[35] Hentschel L., Richter S., Kopp H.G., Kasper B., Kunitz A., Grünwald V., Kessler T., Chemnitz J.M., Pelzer U., Schuler U. (2020). Quality of life and added value of a tailored palliative care intervention in patients with soft tissue sarcoma undergoing treatment with trabectedin: A multicentre, cluster-randomised trial within the German Interdisciplinary Sarcoma Group (GISG). BMJ Open.

[36] Eisenhauer E.A., Therasse P., Bogaerts J., Schwartz L.H., Sargent D., Ford R., Dancey J., Arbuck S., Gwyther S., Mooney M. (2009). New response evaluation criteria in solid tumours: Revised RECIST guideline (version 1.1). Eur. J. Cancer.

[37] Blay J.Y., Italiano A., Ray-Coquard Ι., Le Cesne A., Duffaud F., Rios M., Collard O., Bertucci F., Bompas E., Isambert N. (2013). Long-term outcome and effect of maintenance therapy in patients with advanced sarcoma treated with trabectedin: An analysis of 181 patients of the French ATU compassionate use program. BMC Cancer.

[38] Van Glabbeke M., Verweij J., Judson I., Nielsen O.S. (2002). Progression-free rate as the principal end-point for phase II trials in soft-tissue sarcomas. Eur. J. Cancer.

[39] Demetri G.D., von Mehren M., Jones R.L., Hensley M.L., Schuetze S., Elias A.D., Pierson R.F., Knoblauch R.E., Park Y.C., Wang G.C. (2016). Patient-reported outcomes from randomized, phase-3 study of trabectedin (T) vs. dacarbazine (D) in advanced leiomyosarcoma (LMS) or liposarcoma (LPS). J. Clin. Oncol..

[40] Gough N., Koffman J., Ross J.R., Riley J., Judson I. (2017). Symptom Burden in Advanced Soft-Tissue Sarcoma. J. Pain Symptom Manag..

[41] Gough N.J., Smith C., Ross J.R., Riley J., Judson I. (2011). Symptom burden, survival and palliative care in advanced soft tissue sarcoma. Sarcoma.

