# Supplementary figures and images for: A Multicenter, Prospective, Observational Study to Assess the Clinical Activity and Impact on Symptom Burden and Patients’ Quality of Life in Patients with Advanced Soft Tissue Sarcomas Treated with Trabectedin in a Real-World Setting in Greece
Source: Cancers (Basel). 2022 Apr 8;14(8):1879. doi: 10.3390/cancers14081879 (PMC9031293; doi:10.3390/cancers14081879)

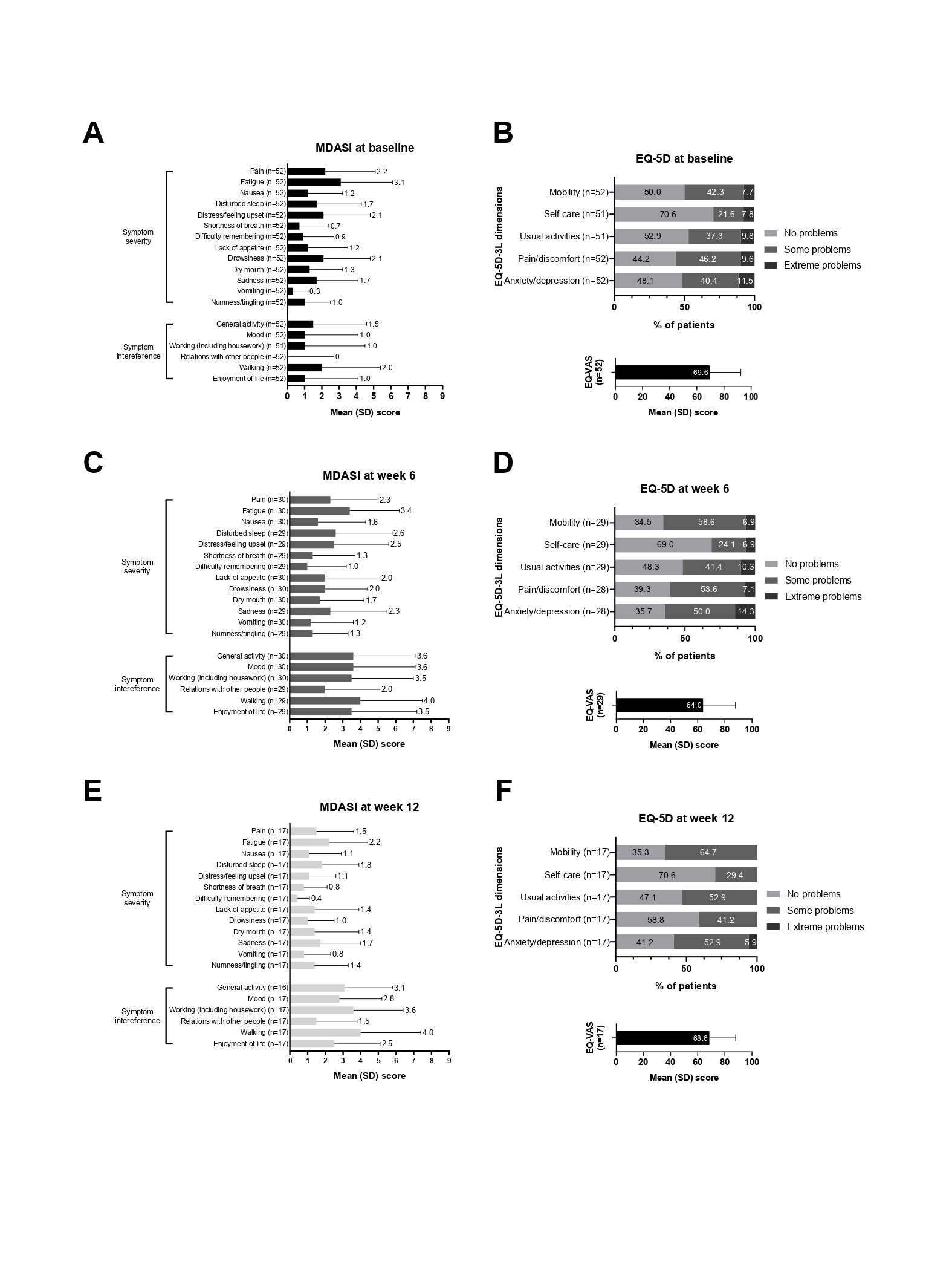

Supplement: Supplementary file 1 [file cancers-14-01879-s001.zip › cancers-1540490-supplementary.jpg]
